# *Polygonum aviculare L*. extract and quercetin attenuate contraction in airway smooth muscle

**DOI:** 10.1038/s41598-018-20409-x

**Published:** 2018-02-15

**Authors:** Xi Luo, Lu Xue, Hao Xu, Qing-Yang Zhao, Qian Wang, Yu-Shan She, Dun-An Zang, Jinhua Shen, Yong-Bo Peng, Ping Zhao, Meng-Fei Yu, Weiwei Chen, Li-Qun Ma, Shu Chen, Shanshan Chen, Xiangning Fu, Sheng Hu, Xiaowei Nie, Chenyou Shen, Chunbin Zou, Gangjian Qin, Jiapei Dai, Guangju Ji, Yunchao Su, Shen Hu, Jingyu Chen, Qing-Hua Liu

**Affiliations:** 1 Institute for Medical Biology and Hubei Provincial Key Laboratory for Protection and Application of Special Plants in Wuling Area of China, College of Life Sciences, South-Central University for Nationalities, Wuhan, 430074 China; 2 Jiangsu Key Laboratory of Organ Transplantation, Department of Cardiothoracic Surgery, Lung Transplant Group, Wuxi People’s Hospital, Nanjing Medical University, Wuxi, Jiangsu China; 3 Department of Cardiovascular Surgery, Union Hospital, Tongji Medical College, Huazhong University of Science and Technology, Wuhan, 430032 Hubei China; 4 Department of Thoracic, Tongji Hospital, Tongji Medical College, Huazhong University of Science and Technology, Wuhan, 430032 Hubei China; 5 Department of Medical Oncology, Hubei Cancer Hospital, Wuhan, 430079 Hubei China; 6 Acute Lung Injury Center of Excellence, Division of Pulmonary, Allergy, and Critical Care Medicine, Department of Medicine, University of Pittsburgh School of Medicine., Pittsburgh, PA 15213 USA; 7 Department of Biomedical Engineering, School of Medicine & School of Engineering, University of Alabama Birmingham, Birmingham, AL 35294 USA; 8 Wuhan Institute for Neuroscience and Engineering, South-Central University for Nationalities, Wuhan, 430074 China; 9 National Laboratory of Biomacromolecules, Institute of Biophysics, Chinese Academy of Sciences, Beijing, 100101 China; 10 Department of Pharmacology and Toxicology, Medical College of Georgia, Augusta University, Augusta, GA 30912 USA; 11 Jonsson Comprehensive Cancer Center, University of California, Los Angeles, CA 90095 USA

## Abstract

Because of the serious side effects of the currently used bronchodilators, new compounds with similar functions must be developed. We screened several herbs and found that *Polygonum aviculare L*. contains ingredients that inhibit the precontraction of mouse and human airway smooth muscle (ASM). High K^+^-induced precontraction in ASM was completely inhibited by nifedipine, a selective blocker of L-type voltage-dependent Ca^2+^ channels (LVDCCs). However, nifedipine only partially reduced the precontraction induced by acetylcholine chloride (ACH). Additionally, the ACH-induced precontraction was partly reduced by pyrazole-3 (Pyr3), a selective blocker of TRPC3 and stromal interaction molecule (STIM)/Orai channels. These channel-mediated currents were inhibited by the compounds present in *P*. *aviculare* extracts, suggesting that this inhibition was mediated by LVDCCs, TRPC3 and/or STIM/Orai channels. Moreover, these channel-mediated currents were inhibited by quercetin, which is present in *P*. *aviculare* extracts. Furthermore, quercetin inhibited ACH-induced precontraction in ASM. Overall, our data indicate that the ethyl acetate fraction of *P*. *aviculare* and quercetin can inhibit Ca^2+^-permeant LVDCCs, TRPC3 and STIM/Orai channels, which inhibits the precontraction of ASM. These findings suggest that *P*. *aviculare* could be used to develop new bronchodilators to treat obstructive lung diseases such as asthma and chronic obstructive pulmonary disease.

## Introduction

Worldwide, approximately 300 million individuals have asthma^1^, and 328.6 million individuals suffer from chronic obstructive pulmonary disease (COPD)^2^. These diseases are typically treated using bronchodilators^3^. Nevertheless, these treatment modalities can cause various side effects, including palpitation, tremor, headache, cardiovascular disease, ischemic heart disease, cardiac failure and death from life-threatening asthma attacks^4,5^, and they can also lead to desensitization^6^. Therefore, new bronchodilators must be developed.

*Polygonum aviculare L*. (*P*. *aviculare*), a member of the Polygonaceae family, is distributed across Asia, Africa, Latin America and the Middle East, where it is used as a traditional medicine. It has antimicrobial^7^ and anti-inflammatory activities^8^. However, we found that the ethyl acetate fraction of *P*. *aviculare* (EAF) can inhibit the precontraction of airway smooth muscle (ASM).

ASM contraction is triggered by a complex pathway^9–13^ involving the increase in intracellular Ca^2+^ and sensitization of Ca^2+^, remodeling of the cytoskeleton and interaction between filaments, and activation of signal pathways. An increase in Ca^2+^ results from Ca^2+^ release mediated by inositol 1,4,5-trisphosphate receptors (IP_3_Rs) and/or ryanodine receptors (RyRs) in the endoplasmic reticulum (ER) membrane and Ca^2+^ influx mediated by Ca^2+^-permeant ion channels in the plasma membrane. Ca^2+^ binds with calmodulin to form a Ca^2+^-calmodulin complex, which then activates myosin light-chain kinase (MLCK). The activated MLCK phosphorylates myosin light chain (LC20) to induce cross-bridge cycling, which results in the contraction of muscle cells.

Ca^2+^ release is transient, and thus it can trigger only a transient contraction. However, release-induced depletion of stored Ca^2+^ activates ion channels that induce a sustained contraction, which can be stimulate by acetylcholine chloride (ACH) and high levels of K^+^. It is known that high levels of K^+^ result in membrane depolarization that activates L-type voltage-dependent Ca^2+^ channels (LVDCCs), which mediate Ca^2+^ influx to trigger a sustained contraction. These channels can also be activated following the stimulation of muscarinic (M) receptors by ACH or methacholine (Mch). In addition, ACH can induce the activation of TRPC3 and stromal interaction molecule (STIM)/Orai channels^14,15^. These channels are permeable to Ca^2+^ and can mediate Ca^2+^ influx to cause sustained contraction. Our results suggest that these Ca^2+^-permeant channels can be inhibited by EAF, which results in the inhibition of the sustained contraction of ASM.

## Results

### Effects of EAF on the contraction of mouse tracheal ASM

**Figure 1:**
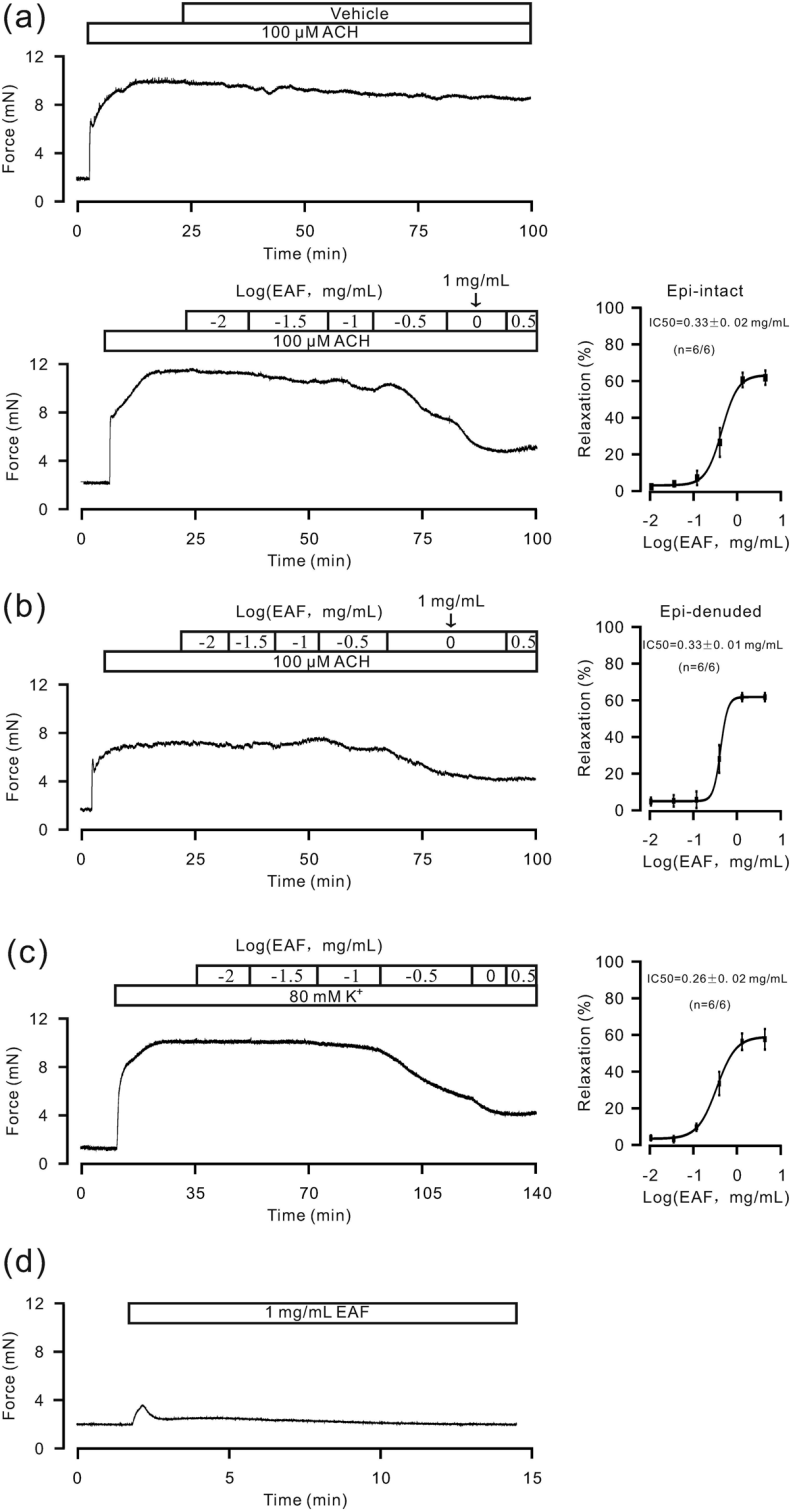
Effects of EAF on mouse tracheal smooth muscle contraction force. (**a**) ACH (100 μM) induced sustained contractions in mouse TRs. The effects of EAF and vehicle were observed. EAF induced relaxation, while the vehicle did not. (**b**) The same experiments were repeated in epithelium-denuded TRs. (**c**) Similar inhibitory effects of EAF on high K^+^-induced contraction were observed in TRs. (**d**) EAF induced a small contraction in resting TRs. These data indicate that EAF attenuates precontraction in ASM and it does not increase the resting tension of ASM.

We first studied the effects of EAF on precontracted ASM induced by ACH and high K^+^ levels (i.e., high-K^+^ solutions containing 80 mM KCl). As shown in Fig. 1a and b, 100 μM ACH induced sustained precontractions in mouse tracheal rings (TRs). The effects of the vehicle (0.1% DMSO) and EAF were then compared. The vehicle did not affect the precontraction in the 6 TRs studied, whereas EAF inhibited TR precontraction in a dose-dependent manner. The half maximal inhibitory concentration (IC_50_) of EAF was 0.33 ± 0.02 mg/mL (n = 6/6, the number of samples/the number of animals), and the maximal inhibition was 59.8 ± 4.7%. These results indicate that EAF potently attenuates ASM precontraction. However, the petroleum ether, chloroform, butyl alcohol and water fractions of *P*. *aviculare* had no such inhibitory effects.

This inhibitory effect of EAF was also observed in epithelium-denuded TRs (Fig. 1b). The removal of the epithelium was confirmed in tissue slices stained with hematoxylin and eosin (data not shown). The IC_50_ value was 0.33 ± 0.01 mg/mL, and the peak inhibition was 61.2 ± 2.2% (n = 6/6). These results show that EAF inhibits the ACH-induced precontraction of epithelium-denuded TRs. Although the dose-inhibition curve was steeper for these samples than that in samples with intact samples, the maximal inhibition was similar. Thus, intact samples were used in subsequent experiments unless otherwise stated.

Additionally, EAF reduced high K^+^-induced precontraction of TRs (Fig. 1c). The IC_50_ was calculated as 0.26 ± 0.02 mg/mL (n = 6/6), with a peak inhibition of 55.9 ± 4.7%.

Moreover, EAF induced very small transient and moderate sustained contractions in quiescent TRs (Fig. 1d). The peak and sustained forces were 1.1 ± 0.06 mN and 0.15 ± 0.07 mN (n = 6/6), respectively. These data suggest that EAF does not induce substantial contraction in resting tracheal ASM.

### EAF reduces the precontraction of mouse bronchial ASM

We further studied the effects of EAF on precontraction in bronchial ASM by measuring the airway lumen area (ALA) in mouse lung slices. As shown in Figure S1, 100 μM ACH decreased the ALA. EAF was then added, and the ALA, measured after 30 min, was markedly higher than that in the vehicle control-treated samples. These data indicate that EAF inhibits precontraction in bronchial ASM.

### EAF decreases the precontraction of human bronchial ASM

**Figure 2:**
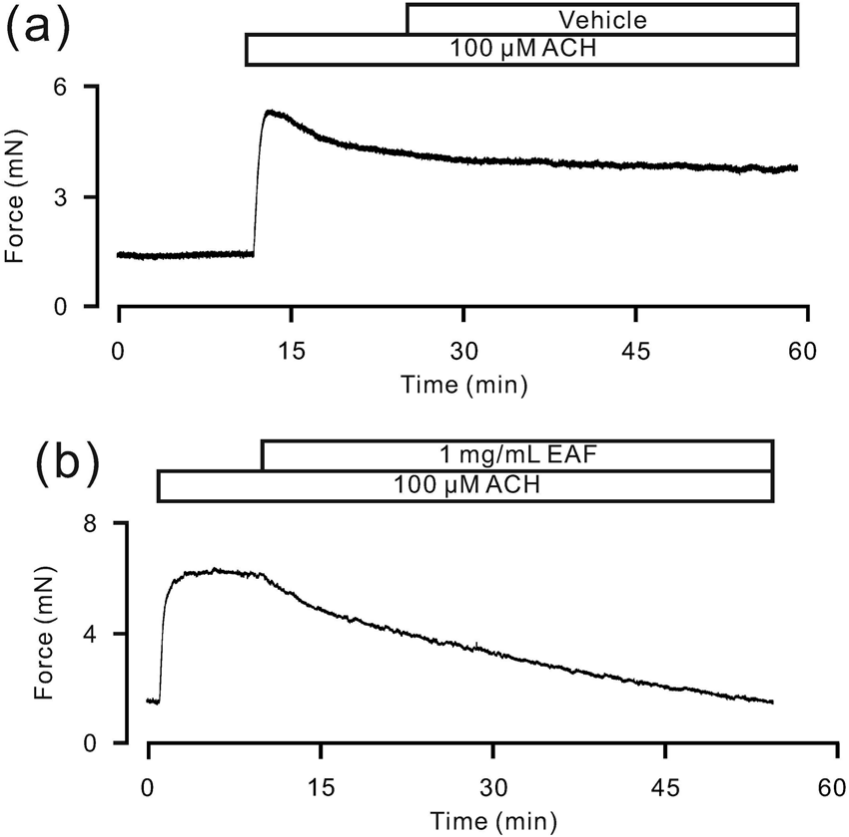
EAF attenuates ACH-induced contraction in human bronchial ASM. ACH was used to induce contractions in human bronchial ASM strips, which were markedly inhibited by EAF but not by the vehicle.

Strips of human bronchial ASM were induced to contract by the addition of ACH, and the effects of vehicle and EAF were observed (Fig. 2). EAF induced a marked relaxation in human bronchial ASM (96.1 ± 2.0%, n = 6 samples/6 subjects), whereas the vehicle had no such effect (n = 6 samples/3 subjects). These results indicate that EAF inhibits ACH-induced precontraction in human bronchial ASM.

### Mechanism of high K^+^- and ACH-induced precontraction

**Figure 3:**
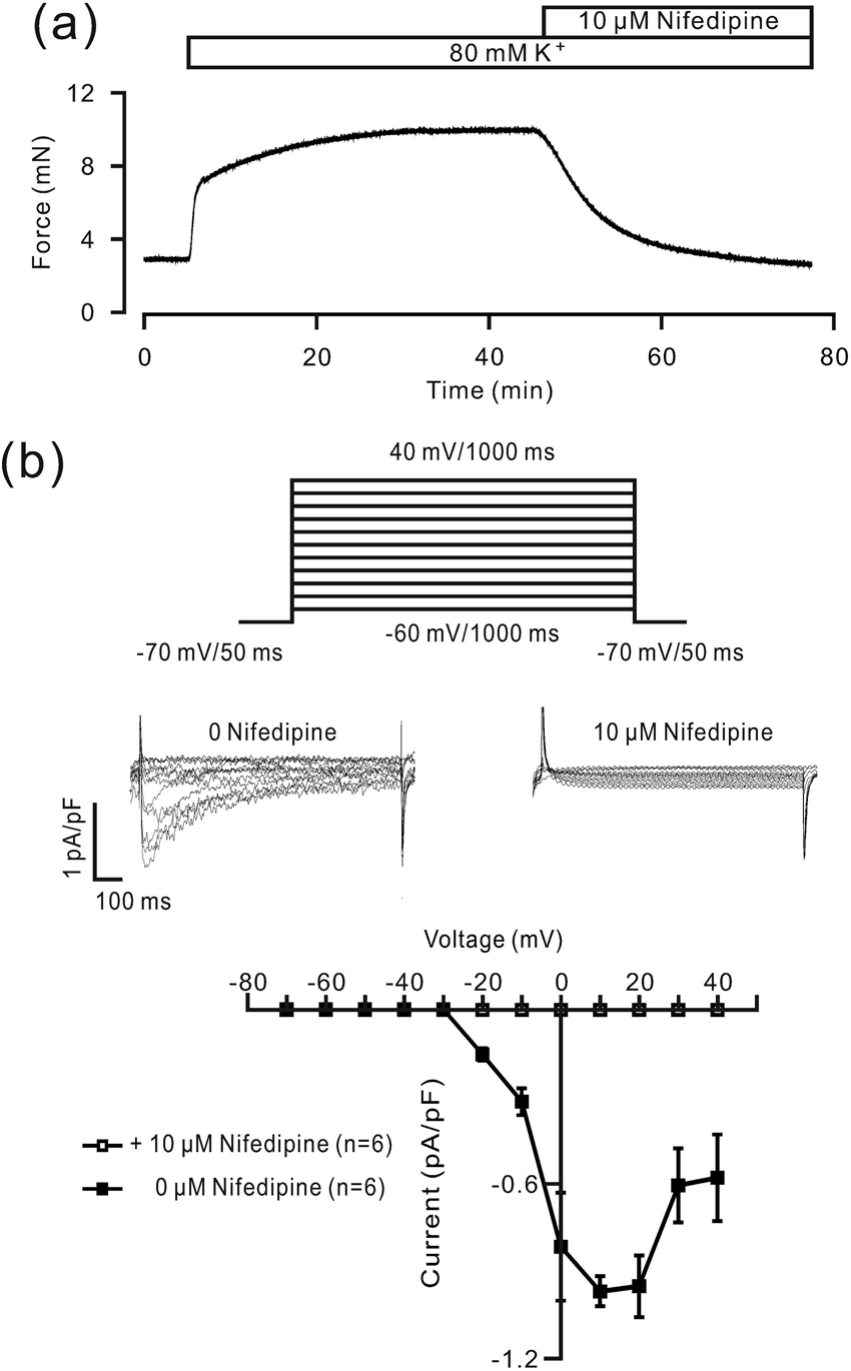
Nifedipine blocks LVDCC-mediated contraction and current. (**a**) A high concentration of K^+^ (80 mM) induced a contraction in a mouse TR, which was almost blocked by the LVDCC inhibitor nifedipine. (**b**) The protocol used to record the LVDCC-mediated current in single mouse tracheal ASM cells (*top*). The current was completely blocked by nifedipine (*middle*). Current-voltage curves (*bottom*). These results indicate that high K^+^-induced contraction might be completely mediated by LVDCCs.

We then investigated the underlying mechanism of precontraction in mouse tracheal ASM induced by high K^+^ levels and ACH. It is known that high K^+^ levels induce plasma membrane depolarization and activate LVDCCs. The activated LVDCCs mediate Ca^2+^ influx, which then triggers contraction. As shown in Fig. 3a, the TR showed a sustained contraction following the addition of high-K^+^ solution, which was almost completely inhibited by 10 μM nifedipine, a selective blocker of LVDCCs. The maximal inhibition was 99.8 ± 0.63% (n = 8/8). Moreover, single tracheal ASM cells exhibited LVDCC-mediated currents, which were completely blocked by nifedipine (Fig. 3b). These results suggest that the high K^+^ level-induced contraction is mediated by LVDCC-mediated Ca^2+^ influx.

**Figure 4:**
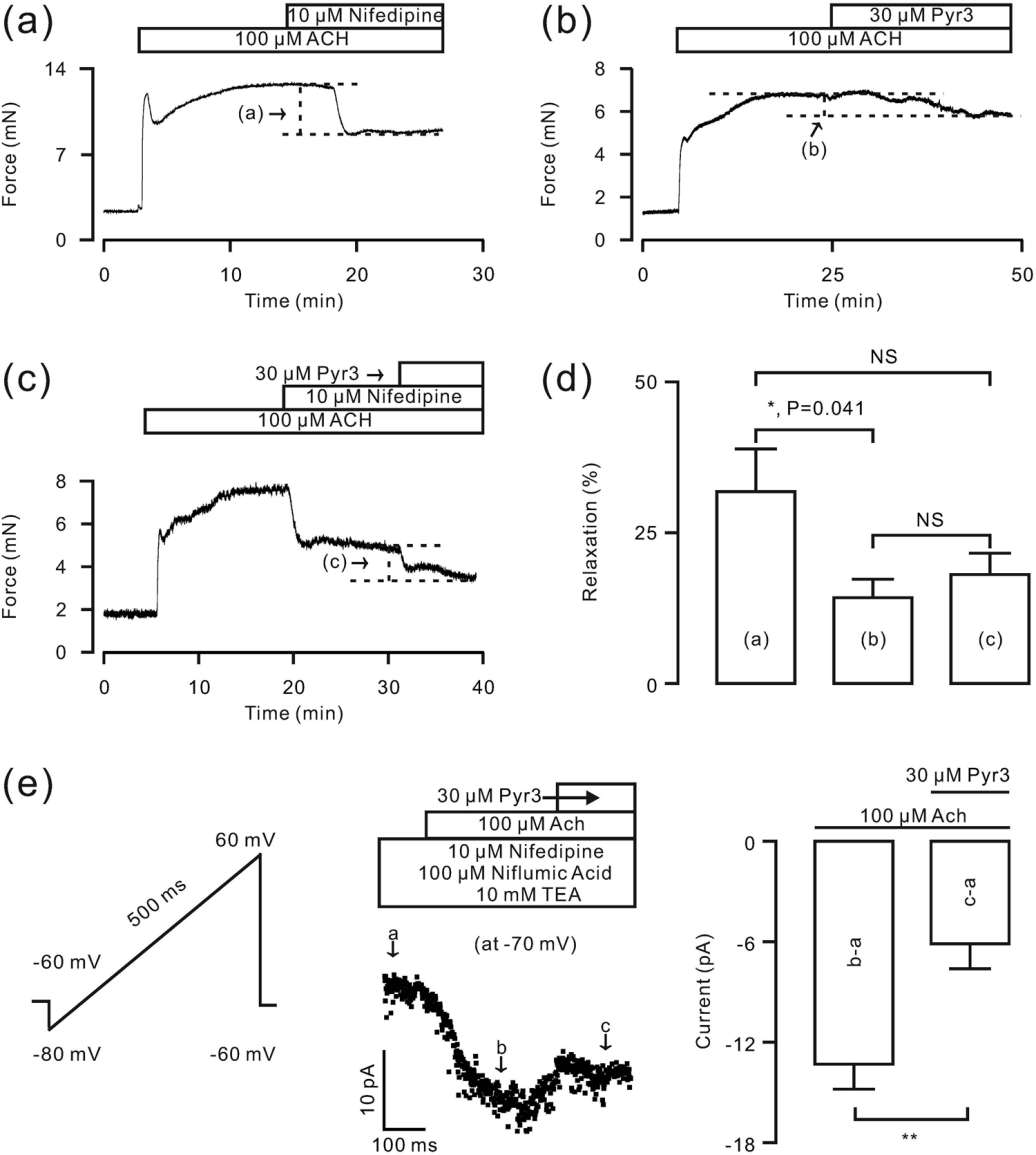
ACH-induced contractions in mouse TRs are inhibited by nifedipine and Pyr3. (**a**–**c**) ACH (100 μM) induced contractions in TRs, which were inhibited by nifedipine and/or Pyr3. (**d**) Summary of results. *P < 0.05. (**e**) In the presence of nifedipine, Pyr3 and NA, ACH-induced NSCC-mediated currents were recorded using a ramp. The values at −70 mV were extracted and used to plot a current-time trace, showing that Pyr3 partially reduced the ACH-induced current. These data suggest that LVDCCs, TRPC3 and/or STIM/Orai channels contribute to the ACH-induced contraction of mouse TRs.

ACH-induced contractions have been reported to be mediated by LVDCCs^14,15^, TRPC3 and/or STIM/Orai channels^15,16^. We found that the LVDCC blocker nifedipine (Fig. 4a) and the TRPC3 and STIM/Orai channel blocker pyrazole-3 (Pyr3)^17^ (Fig. 4b) inhibited the ACH-induced precontraction of TRs. These results were further confirmed by the inhibition of ACH-induced precontraction by the sequential administration of nifedipine and Pyr3 (Fig. 4c). The summary of results show that the extent of nifedipine-induced relaxation (31.8 ± 7.1%, n = 7, Fig. 4d, *bar “a”*) was larger than that caused by Pyr3 (18.1 ± 3.6%, n = 7, Fig. 4d, *bar “b”*); however, the Pyr3-induced relaxation was not different in the absence (Fig. 4d, *bar “b”*) or presence of nifedipine (14.2 ± 3.1%, n = 7, Fig. 4d, *bar “c”*), suggesting that LVDCCs, TRPC3 and STIM/Orai channels partially contribute to ACH-induced precontraction. The role of TRPC3 and STIM/Orai channels in ASM precontraction was further investigated. We used nifedipine, tetraethylammonium chloride (TEA) and niflumic acid (NA) to block LVDCC-mediated and K^+^- and Cl^−^-channel-mediated currents, respectively^15^, because these channels are activated by ACH. The measured currents were thus ACH-activated non-selective cation channel (NSCC)-mediated currents, recorded using a ramp (Fig. 4e). Values at −70 mV were measured and used to plot a current-time trace, showing that Pyr3 reduced the NSCC-mediated currents from −13.3 ± 1.5 pA to −6.1 ± 0.6 pA (P < 0.01). These data suggest that ACH activates TRPC3 and/or STIM/Orai channels.

### Mechanism of EAF-induced inhibition of high K^+^- and ACH-induced precontraction

**Figure 5:**
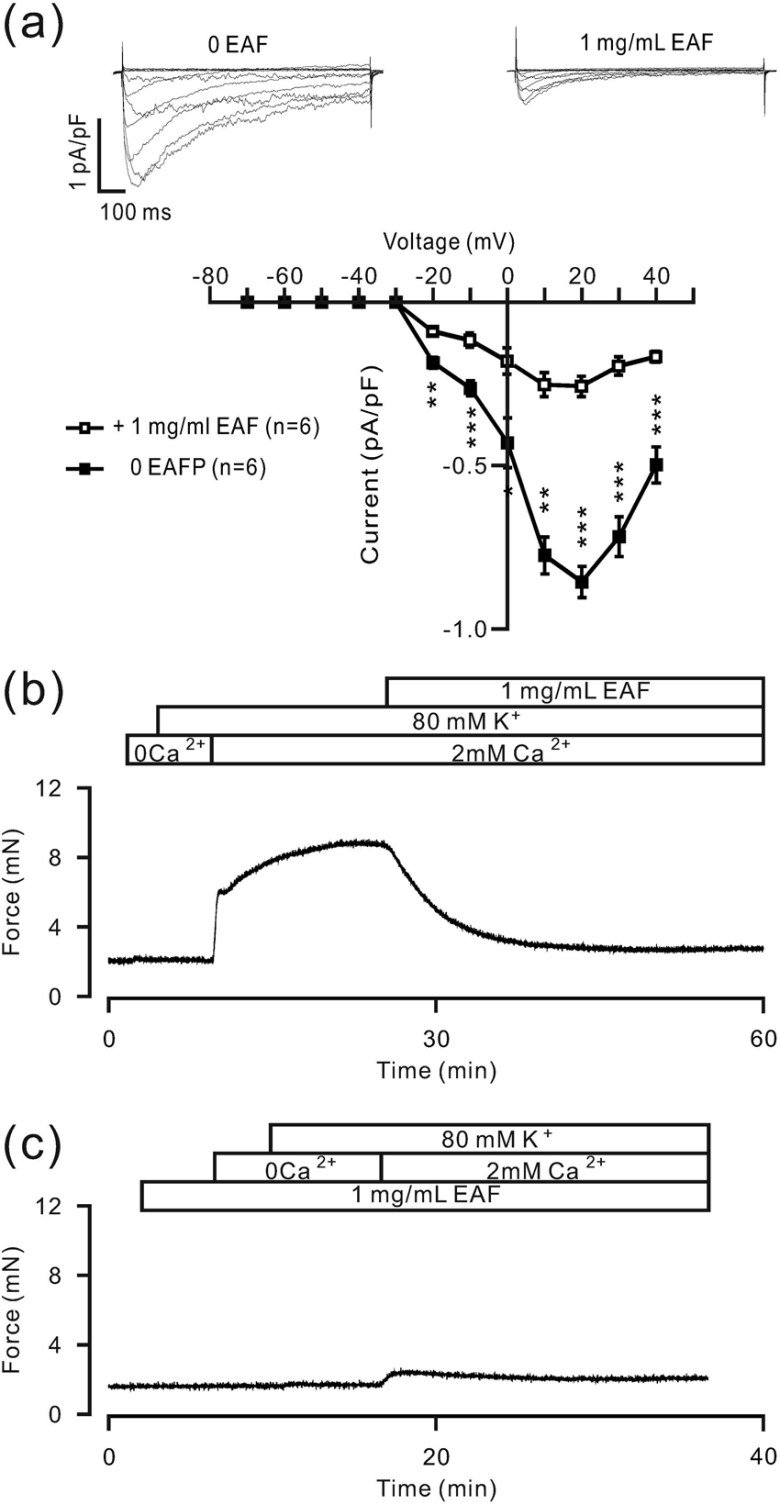
EAF reduces LVDCC-mediated current and TR contraction. (**a**) The LVDCC-mediated currents were recorded as shown in Fig. 3b. The currents were partially inhibited by EAF. The current-voltage curves were plotted. P-values evaluated with paired *t-test* from *left* to *right* are 1.6E-3, 1.8E-4, 1.2E-3, 1.3E-5, 6.1E-5, 9.7E-5 and 1.2E-3 between the currents before and after EAF administration. **P < 0.01; ***P < 0.001. (**b**) Under Ca^2+^-free conditions (0 Ca^2+^  + 0.5 mM EGTA), 80 mM K^+^ failed to induce contractions. However, upon the addition of 2 mM Ca^2+^, contractions were observed, which were markedly inhibited by 1 mg/mL EAF. (**c**) When TRs were incubated with EAF, contractions induced by 2 mM Ca^2+^ were substantially inhibited. These data indicate that the EAF-induced relaxation may be due to its inhibition of LVDCC-mediated Ca^2+^ influx.

We then investigated whether EAF inhibits the pathways defined in the above experiments to promote relaxation. As shown in Fig. 5a, the LVDCC-mediated currents were markedly reduced by EAF, suggesting that EAF potently inhibits LVDCCs. This finding was further supported by the following experiments. Under Ca^2+^-free conditions, 80 mM K^+^ resulted in the opening of LVDCCs, but no contraction occurred, suggesting that the high K^+^-induced contraction is completely dependent on Ca^2+^ influx via LVDCCs. The addition of 2 mM Ca^2+^ induced a contraction, which was reduced to 79.8 ± 4.9% by EAF (n = 6/6); the remaining contraction force was 1.95 ± 0.55 mN (Fig. 5b). These results indicate that EAF inhibits LVDCC-mediated Ca^2+^ influx, and this results in a marked relaxation of ASM. As shown in Fig. 5c, EAF reduced the force of contraction induced by the addition of 2 mM Ca^2+^ (the peak force was 0.91 ± 0.29 mN, n = 6/6; P > 0.05 versus the remaining contraction force in Fig. 5b), further supporting that EAF inhibits LVDCCs.

**Figure 6:**
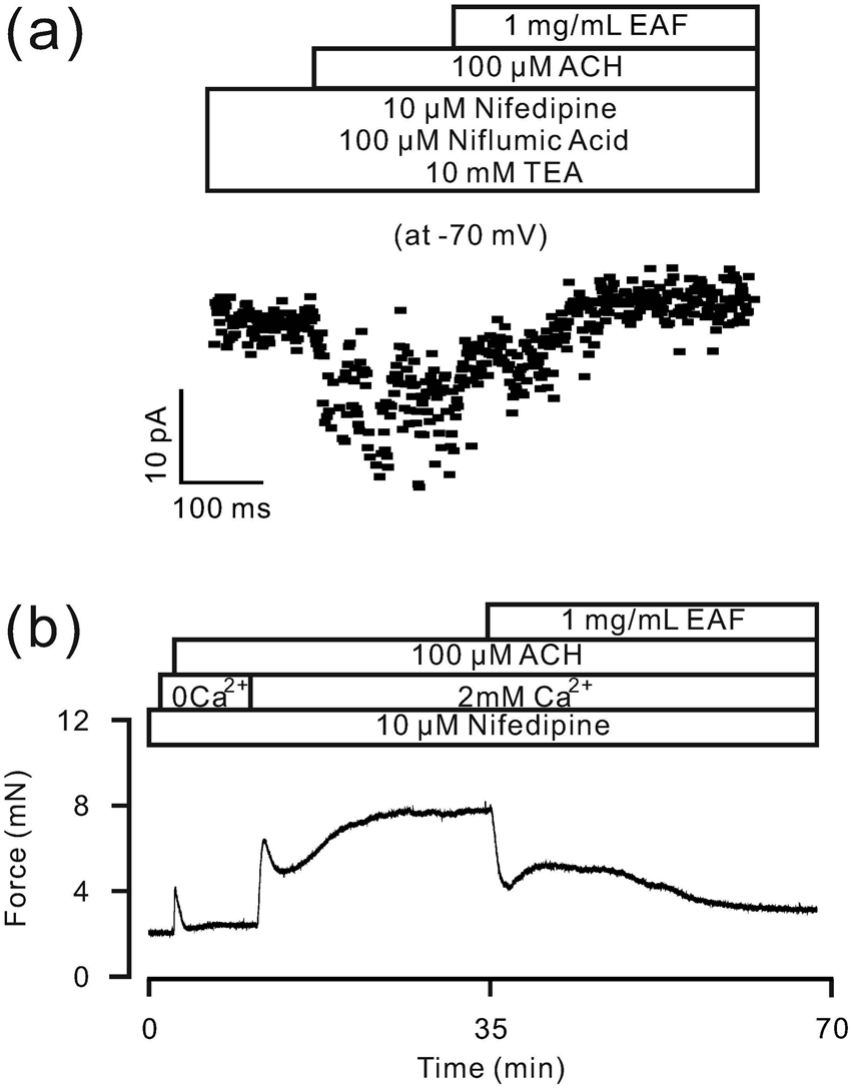
EAF attenuates ACH-induced current and TR contraction. (**a**) ACH-induced currents, similarly recorded as shown in Fig. 4E, were completely blocked by EAF. (**b**) Under Ca^2+^-free conditions (0 Ca^2+^  + 0.5 mM EGTA), ACH induced transient contractions in a TR incubated in nifedipine. After the addition of 2 mM Ca^2+^, a large sustained contraction was observed in the TR, which was attenuated by EAF. These results indicate that TRPC3 and/or STIM/Orai channels are likely blocked by EAF and contribute to EAF-induced relaxation.

We next investigated whether EAF inhibits TRPC3 and STIM/Orai channels. As shown in Fig. 6a, ACH-induced NSCC-mediated currents were similarly isolated using nifedipine, TEA and NA. These currents were completely blocked by EAF (from −14.70 ± 0.56 pA to 0.80 ± 0.11 pA, n = 6), indicating that EAF blocks ACH-activated NSCCs. This finding suggests that EAF blocks TRPC3 and STIM/Orai channels, which are activated by ACH (Fig. 4e).

TRPC3 and STIM/Orai channels are Ca^2+^-permeant NSCCs. As shown in Fig. 6b, ACH induced transient contractions in TRs (1.98 ± 0.17 mN, n = 7/7) under Ca^2+^-free conditions (0 Ca^2+^  + 0.5 mM EGTA) and LVDCC blockade (with nifedipine) due to Ca^2+^ release from the ER. When the contraction reached a plateau, 2 mM Ca^2+^ was added, and additional contractions occurred. These contractions were further blocked by EAF (60.9 ± 3.4%, n = 7/7), suggesting that the blockade of TRPC3 and STIM/Orai channels can contribute to the EAF-induced relaxation of TRs.

### EAF contains quercetin

We then attempted to identify the compound in EAF that plays the role of the relaxant. Quercetin can be extracted from *P*. *aviculare*^18^ and has been shown to relax ASM^19–22^. Therefore, we fractionated EAF using HPLC (Figure S2). The results showed that one peak of EAF overlapped with that of quercetin, indicating that EAF contains quercetin.

### Quercetin inhibits precontraction in mouse tracheal ASM

**Figure 7:**
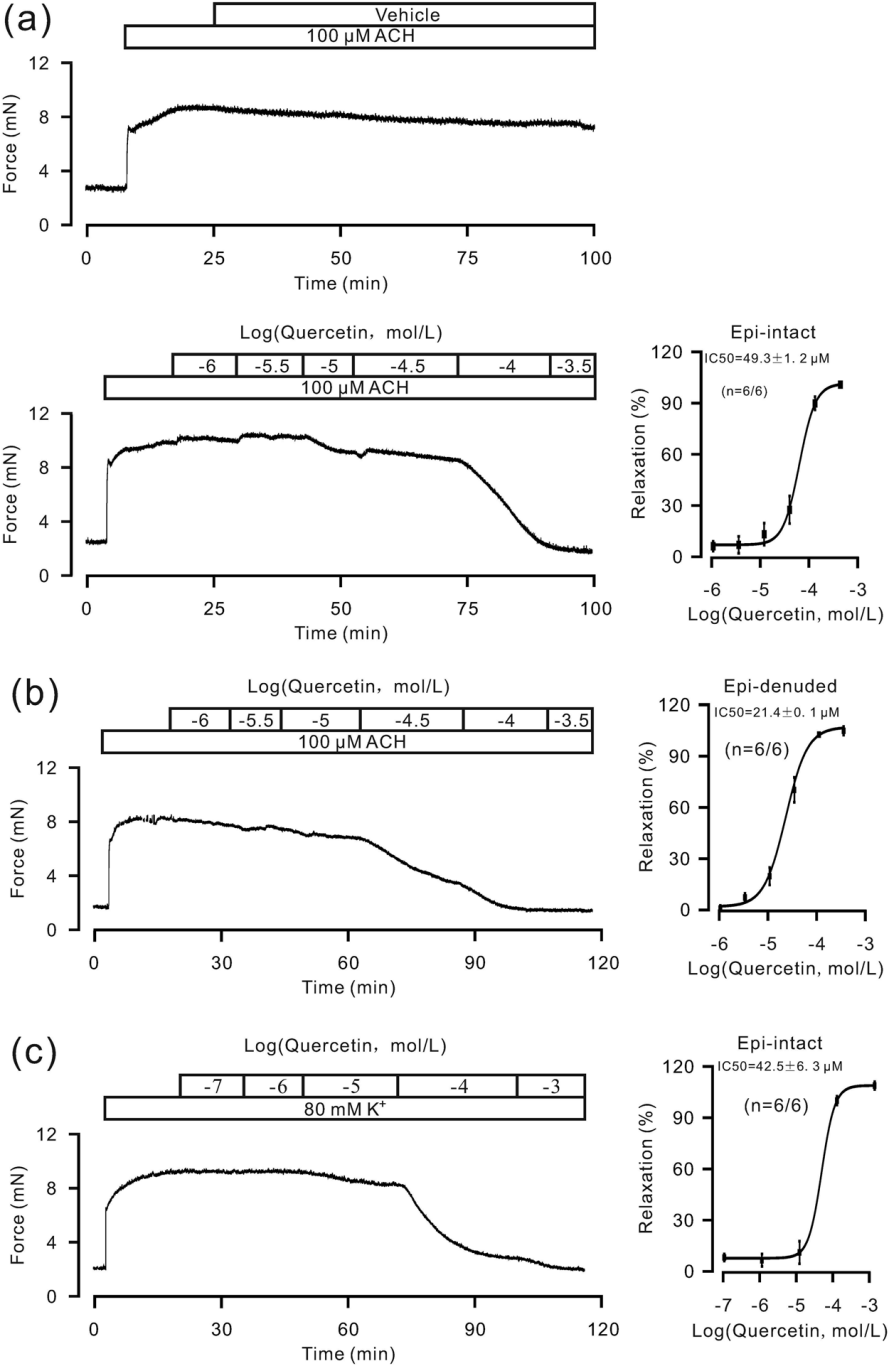
ACH-induced TR contractions are inhibited by quercetin. (**a**,**b**) Quercetin markedly inhibited ACH-induced precontraction in mouse TRs with or without epithelium, but the vehicle had no such effect. (**c**) Quercetin had a similar inhibitory effect on high K^+^-induced contraction in TRs.

We next tested whether quercetin can induce relaxation in precontracted mouse TRs. The quercetin standard induced a substantial relaxation of ACH-induced precontraction in TRs compared to the vehicle (Fig. 7a). The maximal inhibition of quercetin was 104.4 ± 1.1%, and the IC_50_ was 49.3 ± 1.2 μM (n = 6/6). The vehicle failed to inhibit the precontraction of ASM. In addition, the inhibition in epithelium-denuded TRs was 104.8 ± 2.5%, and the IC_50_ was 21.4 ± 0.1 μM (n = 6/6, Fig. 7b). Moreover, quercetin could inhibit high K^+^-induced precontraction (Fig. 7c). The maximal inhibition was 98.9 ± 2.3%, and the IC_50_ was 42.5 ± 6.3 μM (n = 6/6).

To further determine whether quercetin inhibits LVDCCs and ACH-activated NSCCs, we measured the respective channel-mediated currents in response to quercetin. The results show that quercetin completely blocked the currents mediated by LVDCCs (Figure S3a) and ACH-activated NSCCs in mouse tracheal ASM cells (even when the nifedipine-sensitive LVDCCs, TEA-sensitive K^+^ channels and NA-sensitive Cl^−^-channels were blocked) (Figure S3b). These results indicate that quercetin blocks LVDCCs, TRPC3 and STIM/Orai channels.

We next studied the effects of quercetin on the precontraction of human bronchial ASM. As shown in Figure S4, quercetin had similar inhibitory effects on the ACH-induced precontraction of human bronchial ASM. The maximal inhibition of quercetin was 100.7 ± 3.7% (n = 8 samples/8 subjects). The vehicle had no such effect on ACH-induced precontraction (n = 7/4). These results suggest that quercetin inhibits ACH-induced ASM precontraction.

## Discussion

**Figure 8:**
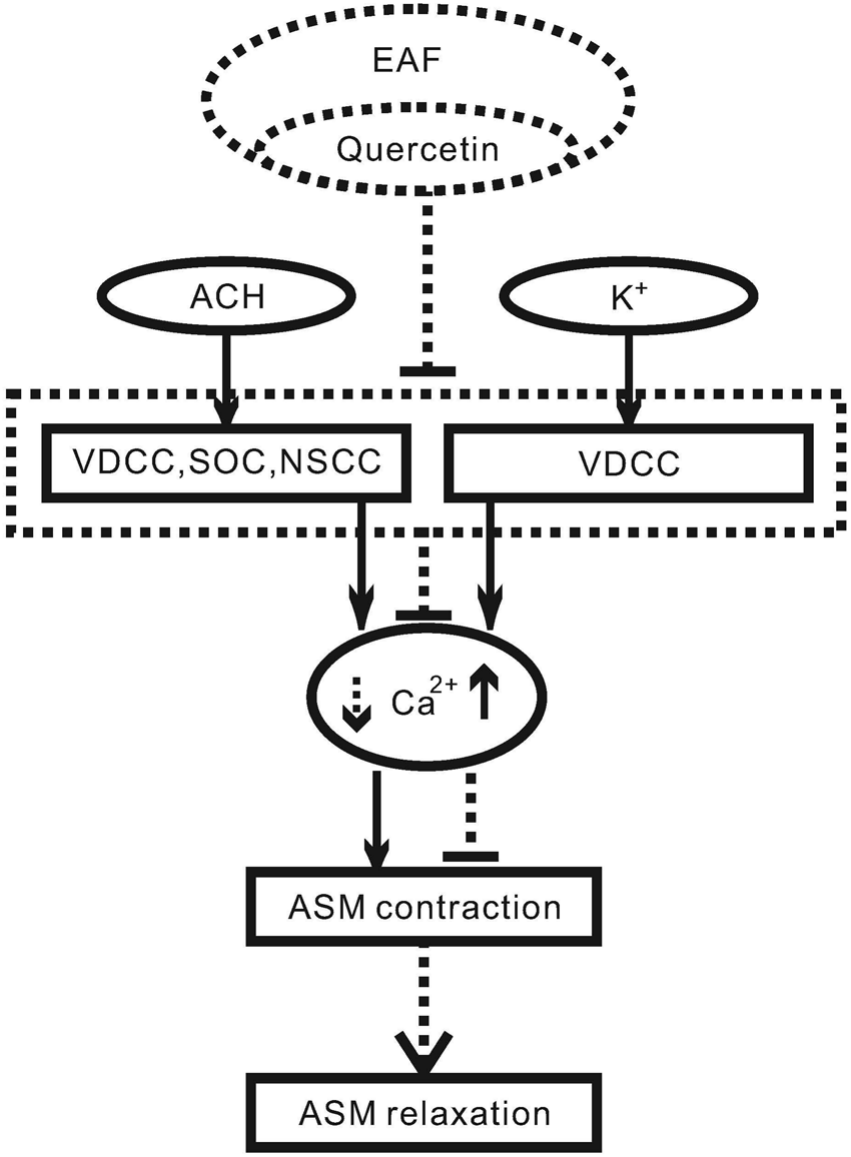
Mechanism of EAF- and quercetin-induced inhibition of TR precontraction. ACH or high levels of K^+^ induces precontractions in ASM by increasing intracellular Ca^2+^ via Ca^2+^-permeant ion channels. The channels are blocked by EAF and quercetin, resulting in a decrease in Ca^2+^, leading to ASM relaxation.

Our results indicate that EAF inhibits high K^+^- and ACH-induced precontraction in ASM. The high K^+^-induced precontraction was completely mediated by LVDCCs; however, the ACH-induced precontraction was at least partially mediated by LVDCCs, TRPC3 and/or STIM/Orai channels. EAF inhibited these channels and thus almost inhibited the precontraction of ASM induced by high K^+^, while it partially attenuated the precontraction of ASM induced by ACH. In addition, EAF was found to contain quercetin. Standard quercetin could also inhibit ASM precontraction and currents mediated by LVDCCs and ACH-activated NSCCs. These results suggest that EAF and quercetin can inhibit ASM precontraction through the inhibition of Ca^2+^-permeant ion channels such as LVDCCs, TRPC3 and STIM/Orai channels (Fig. 8).

We screened active herbal compounds that can inhibit the precontraction of mouse and human ASM induced by ACH or high levels of K^+^ and found that a particular fraction extracted from *P*. *aviculare*, EAF, had this capability (Figs 1, 2 and S1). The epithelial layer of TRs may play a role in the EAF-induced relaxation of TRs, as the dose-inhibition curve was steeper for epithelium-denuded TRs (Fig. 1b) than that for intact control TRs (Fig. 1a). However, the removal of the epithelium did not affect the maximal relaxation induced by EAF; nevertheless, we did not investigate the underlying mechanism much further and used intact samples for most of the experiments in this study. The petroleum ether, chloroform, butyl alcohol and water fractions of *P*. *aviculare* had no effect on the precontraction of ASM. These data indicate that EAF attenuates the precontraction of ASM.

We identified the pathways involved in the EAF-induced inhibition of high K^+^-induced precontraction of ASM. High K^+^-induced contraction is known to be mediated by LVDCCs^15^, and thus, we blocked LVDCCs with nifedipine. The results show that the high K^+^-induced precontraction and LVDCC-mediated currents were almost completely inhibited (Fig. 3a), suggesting that the high K^+^-induced contraction is completely dependent on LVDCC-mediated Ca^2+^ influx. Therefore, EAF likely inhibits the above pathway. To confirm this postulation, we investigated whether EAF blocks LVDCC-mediated currents and observed a partial blockade (Fig. 5a). Additionally, the LVDCC-mediated Ca^2+^ influx-induced contraction was partially inhibited by EAF, which was validated by the marked, albeit incomplete, inhibition of the high K^+^-induced contraction by EAF (Fig. 5b,c). Therefore, the remaining contractile force (Fig. 5b,c) may have been due to the LVDCCs that could not be inactivated. This result was different from that with EAF alone, as EAF was shown to induce only small contractions in resting TRs (Fig. 1d).

We also investigated the underlying mechanism by which EAF inhibits ACH-induced precontraction. LVDCCs, TRPC3 and STIM/Orai channels are known to participate in ACH-induced contraction^15^. Our results indicate that nifedipine and Pyr3 induce a partial relaxation of ASM (Fig. 4a–d), suggesting that LVDCCs, TRPC3 and/or STIM/Orai channels contribute to ACH-induced contraction. As these channels were inhibited by EAF, we concluded that they mediate the EAF-induced relaxation in ASM. In the present study, we wanted to determine whether EAF inhibits these channels and then induces relaxation, and indeed, we found that EAF inhibited LVDCCs and then resulted in relaxation, as described above. In addition, we found that ACH induced currents that could be partially blocked by Pyr3 (Fig. 4e), suggesting that ACH activates TRPC3 and/or STIM/Orai channels. Moreover, the ACH-induced currents were completely inhibited by EAF (Fig. 6a), indicating that EAF could block TRPC3- and/or STIM/Orai channel-mediated currents. Consistent with these results, EAF reduced the Ca^2+^ influx-induced contraction under conditions of LVDCC blockade (Fig. 6b). This Ca^2+^ influx was mediated by TRPC3 and/or STIM/Orai channels (Fig. 4e) and other pathways because Pyr3-resistant currents were still present; however, the actual pathways involved in this process and whether these resistant currents contribute to ASM contraction remain to be defined. Nevertheless, the results suggest that EAF can inhibit LVDCCs, TRPC3 and STIM/Orai channels and thus block ACH-induced precontraction.

*P*. *aviculare* contains quercetin^18^, and quercetin has been shown to induce ASM relaxation^19–22^. Moreover, quercetin was previously shown to inhibit LVDCCs and reduce high K^+^-induced precontraction in rat coronary artery smooth muscle cells^23^. We demonstrated that EAF indeed contained quercetin (Figure S2). Therefore, we investigated the effects of standard quercetin on ASM precontraction along with the underlying mechanism. The results showed that quercetin inhibited ACH- and high K^+^-induced precontraction in mouse TRs (Fig. 7), and it reduced currents mediated by LVDCCs and ACH-activated NSCCs in mouse tracheal ASM cells (Figure S3) and ACH-induced precontraction in human bronchial ASM strips (Figure S4). These results indicate that quercetin and EAF have similar effects on ASM and that the underlying mechanisms may be similar.

We conclude that EAF relaxes precontracted ASM by inhibiting Ca^2+^-permeant LVDCCs, TRPC3 and/or STIM/Orai channels. Quercetin contained in EAF and standard quercetin have similar inhibitory effects on ASM contraction. These findings suggest that *P*. *aviculare* could be used to develop new bronchodilator agents.

## Materials and Methods

### Guideline statement

All methods used in this study are in accordance with protocols approved by the South Central University for Nationalities. All animal studies and experiments were conducted under guidelines and protocols approved by the Institutional Animal Care and Use Committee of the South Central University for Nationalities. Human tissues were obtained with informed consent from unused lung tissues of transplant donors and recipients and from normal segments resected from lung carcinoma patients. All experiments were performed under guidelines and protocols approved by the Ethics Committee of the South Central University for Nationalities.

### Reagents

Nifedipine, NA, tetraethylammonium chloride (TEA), ACH, pyrazole-3 (Pyr3), bovine serum albumin (BSA), papain, collagenase H, dithiothreitol (DTT), dithioerythritol (DTE), cesium acetate, cesium chloride (CsCl), Mg-ATP, and agarose were purchased from Sigma (St. Louis, MO, USA). Nifedipine and Pyr3 were dissolved in dimethyl sulfoxide (DMSO), and other compounds were dissolved in the appropriate solutions used in the experiments.

### Plant material and extraction

*P*. *aviculare* was purchased from Beijing Tongrentang (Wuhan, China) and authenticated by Professor Ding-Rong Wan (College of Pharmacy, South Central University for Nationalities). A voucher specimen was deposited at the herbarium of College of Pharmacy, South Central University for Nationalities.

*P*. *aviculare* plants were dried, powdered and then extracted thrice with 70% aqueous ethyl alcohol. The extracts were boiled for 2.5 h, and the supernatants were collected and dried under reduced pressure. The dried products were mixed with 600 mL water, and the lipids were removed using petroleum ether. The remaining components were then extracted with ethyl acetate and dried under reduced pressure. The final product, EAF, was dissolved in 3% DMSO for all experiments.

### HPLC analysis

HPLC analysis was performed using an Ultimate 3000 instrument (Thermo Fisher Scientific Inc., Waltham, MA, USA). Fractionation was performed using a ReproSil 100 C_18_ column (4.6 × 250 mm, 5 μm; Dr. A. Maisch GmbH, Ammerbuch, Germany) as the stationary phase and methanol:0.1% aqueous phosphoric acid (65:35) as the mobile phase. The flow rate was 1.0 mL/min. The detection wavelength was set at 366 nm.

### Animals

Six-week-old male BALB/c mice were purchased from the Hubei Provincial Center for Disease Control and Prevention (Wuhan, China) and housed in a temperature and humidity controlled room with a 12 h light/12 h dark cycle.

### Measurement of ASM contraction

The contraction of ASM in TRs was measured as previously described with some modifications^15,24–26^. Briefly, mice were sacrificed with intraperitoneal injections of sodium pentobarbital (150 mg/kg). The tracheae were dissected and transferred to ice-cold physiological salt solution (PSS) containing (in mM) NaCl 135, KCl 5, MgCl_2_ 1, CaCl_2_ 2, HEPES 10, and glucose 10 (pH = 7.4, adjusted with NaOH). TRs (~5 mm) were cut from the bottom of the tracheae and mounted in 10-mL organ baths containing PSS bubbled with 95% O_2_ and 5% CO_2_ at 37 °C. A 0.3 g preload was added. The TRs were equilibrated for 60 min and precontracted three times with 10^−4^ M ACH. After 30 min, experiments were performed with high-K^+^ solution containing (in mM) NaCl 60, KCl 80, MgCl_2_ 1, CaCl_2_ 2, HEPES 10, and glucose 10 (pH = 7.4, adjusted with NaOH).

Additionally, ASM contraction was indirectly measured in mouse lung slices^25,27^. Briefly, after the mice were euthanized, the tracheae and lungs were exposed. The tracheae were cannulated. Each lung was slowly inflated with warm agarose solutions (2%, 37 °C, ~1.3 mL) followed by ~0.2 mL of air. The lungs were then cooled at 4 °C to gel the agarose. Single lobes were removed and sectioned into ~140-μm thick slices with a vibratome (VT1000S, Leica, Nussloch, Germany). The slices were collected and transferred to Dulbecco’s modified Eagle’s medium (DMEM) (GIBCO) and maintained in an incubator (with 95% O_2_ and 5% CO_2_ at 37 °C) for 2 h before being used in experiments. The slices were placed in a chamber and held in place with a small nylon mesh. The chamber was placed under an LSM 700 laser confocal microscope (LSM 700, Carl Zeiss, Goettingen, Germany) and was perfused with Hanks’ balanced salt solution (HBSS) at a rate of ∼800 μL/min at room temperature. HBSS was supplemented with 20 mM HEPES (containing in mM, NaCl 137.93, KCl 5.33, NaHCO_3_ 4.17, CaCl_2_ 1.26, MgCl_2_ 0.493, MgSO_4_ 0.407, KH_2_PO 0.4414, Na_2_HPO_4_ 0.338, and D-glucose 5.56; adjusted to a pH of 7.4). Images were acquired at a rate of 30 frames/min under a 10 × objective. ALA was calculated using Zen 2010 software (Carl Zeiss).

ASM contraction was also measured in human bronchial ASM as described above. ASM strips were cut from airways and transversely mounted in chambers. The preload was set at 0.3–0.8 g based on the tissue size.

### Isolation of single ASM cells

Single ASM cells were isolated from BALB/c mouse tracheal smooth muscle as previously described with minor modifications^24,28^. Briefly, after the mice were euthanized, the tracheae were removed and transferred to ice-cold PSS. The tracheal muscle was dissected, minced and incubated for 22 min at 35 °C in low-Ca^2+^ PSS (LCPSS; PSS containing 100 μM Ca^2+^) supplemented with 2 mg/mL papain, 1 mg/mL DTE, and 1 mg/mL BSA. The tissues were then transferred to LCPSS containing 1 mg/mL collagenase H, 0.15 mg/mL DTT, and 1 mg/mL BSA for 8 min at 35 °C. The tissues were washed with LCPSS and then gently triturated to yield single ASM cells. Cells were kept on ice and used for experiments within 4 h.

### Measurement of ion channel-mediated current

Current from LVDCCs and ACH-activated channels was recorded using an EPC-10 patch-clamp amplifier (HEKA, Lambrecht, Germany) at room temperature as previously described with some modifications^29,30^. For LVDCC-mediated current measurement, Ba^2+^ was used as the charge carrier. The pipette solution contained (in mM) CsCl 130, EGTA 10, MgCl_2_ 4, Mg-ATP 4, HEPES 10, and TEA 10 (pH = 7.2, adjusted with CsOH). The bath solution contained (in mM) NaCl 105, CsCl 6, BaCl_2_ 27.5, glucose 11, HEPES 10, TEA 10, and NA 0.1 (pH = 7.4, adjusted with NaOH). The holding potential was -70 mV, and the current was measured every 10 s using 1000-ms steps from -70 to 40 mV in 10 mV increments. For ACH-activated current measurements, the pipette solution contained (in mM) CsCl 18, cesium acetate 108, MgCl_2_ 1.2, HEPES 10, EGTA 3, and CaCl_2_ 1 (pH = 7.2, adjusted with Tris-HCl). The free Ca^2+^ concentration was approximately 70 nM as calculated using the WEBMAXC standard program (http://www.stanford.edu/~cpatton/webmaxc/webmaxcS.htm). The bath solution was PSS without K^+^ containing 10 μM nifedipine, 100 μM NA, and 10 mM TEA to block Ca^2+^-, Cl^−^- and K^+^-channel-mediated current, respectively. Cells were patched in the classical whole-cell configuration and held at −60 mV. The current was measured using a 500-ms ramp from −80 to 60 mV, and the values at −70 mV were used to represent the current.

### Hematoxylin and eosin staining

The depletion of airway epithelia was confirmed by hematoxylin & eosin staining as previously described with some modifications^31^. Briefly, after the experiments were complete, the TRs were fixed using 4% paraformaldehyde for 10 min at room temperature, sectioned into 8-μm slices and then stained with hematoxylin and eosin. The slices were examined under a microscope.

### Statistical analysis

The results are expressed as the mean ± standard error of mean (SEM). Student’s t-test between two groups and one-way ANOVA among multiple groups were performed, and the statistical significance was set at P < 0.05.

## Electronic supplementary material

Supplementary Information
