# Supplementary material for: Polygonum aviculare L. extract and quercetin attenuate contraction in airway smooth muscle
Source: Sci Rep. 2018 Feb 15;8:3114. doi: 10.1038/s41598-018-20409-x (PMC5814568; doi:10.1038/s41598-018-20409-x)
Supplement: Supplementary file 1 — Supplementary Information [file 41598_2018_20409_MOESM1_ESM.pdf]

TITLE

***Polygonum aviculare L.* extract and quercetin attenuate contraction in airway smooth muscle**

AUTHORS AND AFFILIATIONS

Xi Luo[1,†], Lu Xue[1,†], Hao Xu[1], Qing-Yang Zhao[1], Qian Wang[1], Yu-Shan She[1], Dun-An Zang[1], Jinhua Shen[1], Yong-Bo Peng[1], Ping Zhao[1], Meng-Fei Yu[1], Weiwei Chen[1], Li-Qun Ma[1], Shu Chen[3], Shanshan Chen[3], Xiangning Fu[4], Sheng Hu[5], Xiaowei Nie[2], Chenyou Shen[2], Chunbin Zou[6], Gangjian Qin[7], Jiapei Dai[8], Guangju Ji[9], Yunchao Su[10], Shen Hu[11], Jingyu Chen[2,*], Qing-Hua Liu[1,]**

[1]Institute for Medical Biology and Hubei Provincial Key Laboratory for Protection and Application of Special Plants in Wuling Area of China, College of Life Sciences, South-Central University for Nationalities, Wuhan 430074, China;

[2]Jiangsu Key Laboratory of Organ Transplantation, Department of Cardiothoracic Surgery, Lung Transplant Group, Wuxi People's Hospital, Nanjing Medical University, Wuxi, Jiangsu, China;

[3]Department of Cardiovascular Surgery, Union Hospital, Tongji Medical College, Huazhong University of Science and Technology, Wuhan, 430032, Hubei, China;

[4]Department of Thoracic, Tongji Hospital, Tongji Medical College, Huazhong University of Science and Technology, Wuhan, 430032, Hubei, China;

[5]Department of Medical Oncology, Hubei Cancer Hospital, Wuhan, 430079, Hubei, China;

[6]Acute Lung Injury Center of Excellence, Division of Pulmonary, Allergy, and Critical

Care Medicine, Department of Medicine, University of Pittsburgh School of Medicine. Pittsburgh, PA, 15213, USA;

[7]*Department of Biomedical Engineering, School of Medicine & School of Engineering, University of Alabama Birmingham, Birmingham, AL 35294, USA;*

[8]*Wuhan Institute for Neuroscience and Engineering, South-Central University for Nationalities, Wuhan 430074, China;*

[9]*National Laboratory of Biomacromolecules, Institute of Biophysics, Chinese Academy of Sciences, Beijing 100101, China;*

[10]*Department of Pharmacology and Toxicology, Medical College of Georgia, Augusta University, Augusta, GA 30912, USA.*

[11]*Jonsson Comprehensive Cancer Center, University of California, Los Angeles, CA 90095, USA.*

[†]*These authors contributed equally to this work.*

***Correspondence author:*

Qing-Hua Liu, M.D., Ph.D., Institute for Medical Biology, College of Life Sciences, South-Central University for Nationalities, 182 MinZu Ave., Wuhan 430074, Hubei, People's Republic of China. Tel/Fax: 0086-27-67841906; E-mail: liu258q@yahoo.com; qinghualiu@mail.scuec.edu.cn

**Co-correspondence author:*

Jingyu Chen, M.D., Jiangsu Key Laboratory of Organ Transplantation, Department of Cardiothoracic Surgery, Lung Transplant Group, Wuxi People's Hospital, Nanjing Medical University, Wuxi, Jiangsu, China; Tel: 0510-85351886; E-mail:

chenjingyu333@sina.com

Running title: *Polygonum aviculare L.* extract inhibits ASM precontraction

Supplementary figures

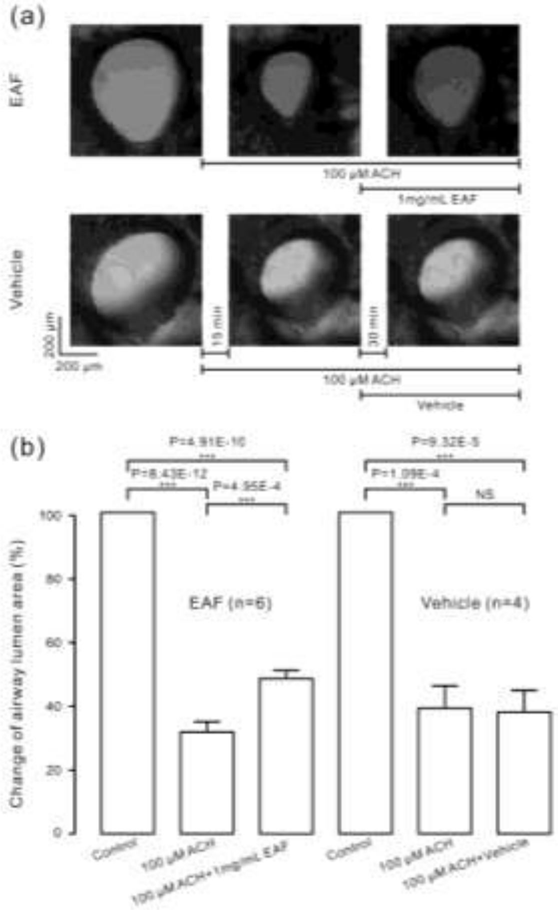

Figure S1. EAF inhibits ACH-induced contraction in mouse bronchial ASM. (a) The airway lumen area (*left*) in lung slices decreased following the addition of ACH (*middle*), which was markedly reversed by EAF (*right/top*) but not by the vehicle (*right/bottom*). **(b)** The summary of results evaluated using a one-way ANOVA with selected post hoc comparisons. NS: P > 0.05; ***: P < 0.001.

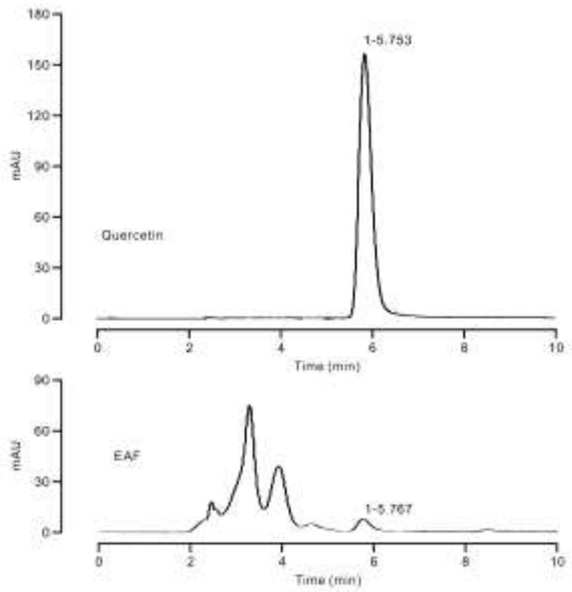

Figure S2. EAF contains quercetin. EAF and the quercetin standard were fractionated via HPLC. One of the peaks of EAF overlapped with that of standard quercetin. These results were observed in 5 independent experiments.

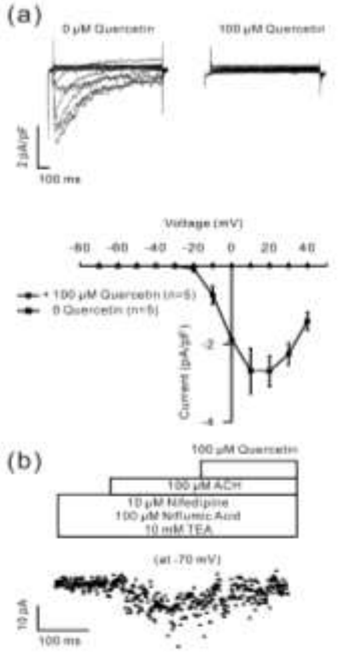

Figure S3. Quercetin inhibits LVDCC-mediated and ACH-activated currents. (a) LVDCC-mediated currents were recorded as shown in Figure 3b and were blocked by quercetin. Current-voltage curves were constructed. **(b)** ACH-induced NSCC-mediated currents, assessed using three inhibitors and special internal and external solutions, were measured as shown in Figure 4e and found to be abrogated by quercetin. These results indicate that quercetin can inhibit current mediated by LVDCCs and ACH-activated NSCCs.

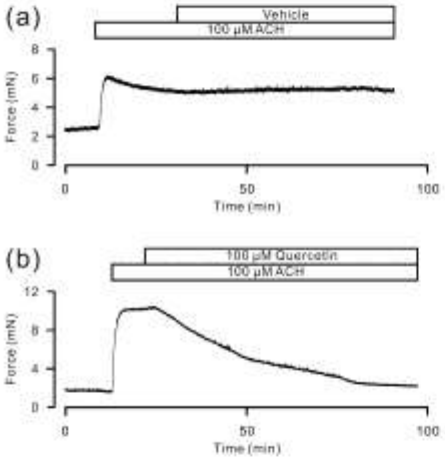

Figure S4. Quercetin inhibits ACH-induced contraction in human bronchial ASM. ACH induced sustained contractions in human bronchial ASM strips, which were inhibited by quercetin but not by the vehicle.
